# The effect of chronic progressive-dose sodium bicarbonate ingestion on CrossFit-like performance: A double-blind, randomized cross-over trial

**DOI:** 10.1371/journal.pone.0197480

**Published:** 2018-05-17

**Authors:** Krzysztof Durkalec-Michalski, Emilia E. Zawieja, Tomasz Podgórski, Igor Łoniewski, Bogna E. Zawieja, Marta Warzybok, Jan Jeszka

**Affiliations:** 1 Institute of Human Nutrition and Dietetics, Poznań University of Life Sciences, Poznań, Poland; 2 Department of Biochemistry, University School of Physical Education in Poznan, Poznań, Poland; 3 Department of Biochemistry and Human Nutrition, Pomeranian Medical University, Szczecin, Poland; 4 Department of Mathematical and Statistical Methods, Poznań University of Life Sciences, Poznań, Poland; Fondazione Toscana Gabriele Monasterio, ITALY

## Abstract

**Background:**

Sodium bicarbonate (SB) has been proposed as an ergogenic aid, as it improves high-intensity and resistance exercise performance. However, no studies have yet investigated SB application in CrossFit. This study examined the effects of chronic, progressive-dose SB ingestion on CrossFit-like performance and aerobic capacity.

**Methods:**

In a randomized, double-blind, cross-over trial, 21 CrossFit-trained participants were randomly allocated to 2 groups and underwent 2 trials separated by a 14-day washout period. Participants ingested either up to 150 mg∙kg^-1^ of SB in a progressive-dose regimen or placebo for 10 days. Before and after each trial, Fight Gone Bad (FGB) and incremental cycling (ICT) tests were performed. In order to examine biochemical responses, blood samples were obtained prior to and 3 min after completing each exercise test.

**Results:**

No gastrointestinal (GI) side effects were reported during the entire protocol. The overall FGB performance improved under SB by ~6.1% (p<0.001) and it was ~3.1% higher compared to post placebo (PLA_post_) (p = 0.040). The number of repetitions completed in each round also improved under SB (mean from baseline: +5.8% to +6.4%). Moreover, in ICT, the time to ventilatory threshold (VT) (~8:25 min SB_post_ vs. ~8:00 min PLA_post_, p = 0.020), workload at VT (~218 W SB_post_ vs. ~208 W PLA_post_, p = 0.037) and heart rate at VT (~165 bpm SB_post_ vs. ~161 bpm PLA_post_, p = 0.030) showed higher SB_post_ than PLA_post_. Furthermore, the maximum carbon dioxide production increased under SB by ~4.8% (from ~3604 mL∙min^-1^ to ~3776 mL∙min^-1^, p = 0.049). Pyruvate concentration and creatine kinase activity before ICT showed higher SB_post_ than PLA_post_ (~0.32 mmol∙L^-1^ vs. ~0.26 mmol∙L^-1^, p = 0.001; ~275 U∙L^-1^ vs. ~250 U∙L^-1^, p = 0.010, respectively). However, the small sample size limits the wide-application of our results.

**Conclusions:**

Progressive-dose SB ingestion regimen eliminated GI side effects and improved CrossFit-like performance, as well as delayed ventilatory threshold occurrence.

## Introduction

In order to allow muscle contraction, adenosine triphosphate (ATP) is used by myosin adenosine triphosphatase (ATPase) resulting in energy production. However, ATP stores are limited and become depleted soon after the onset of exercise, hence requiring ATP resynthesis from other fuel sources such as phosphocreatine, carbohydrates and fats [1]. High-intensity exercise results in the accumulation of lactate and hydrogen ions (H^+^) as a consequence of intensified anaerobic glycolysis when limited amounts of oxygen are available to the working muscle cells [2]. Cellular acidosis leads to a decrease in energy production due to reduction of the proton gradient between mitochondrial matrix and cell cytoplasm [3]. The increasing intracellular acidosis is implicated as one of many factors contributing to the perception of fatigue [4], which is due to the inhibition of key enzymes in energy metabolism [5] as well as the impairment of muscle excitation [6]. Moreover, Abramowitz et al. [7] observed that a lower serum bicarbonate concentration and increased anion gap is associated with reduced cardiorespiratory fitness. The elimination of H^+^ from muscle cells by the buffering systems is pivotal for sustaining muscle contractility [8]. During high-intensity exercise, intramuscular acidity is regulated by intracellular, extracellular and dynamic buffering [9], and bicarbonate (HCO_3_^-^) in particular is a major contributor to the buffering system in blood [10]. In theory, increasing HCO_3_^-^ concentrations could augment high-intensity performance. Therefore, sodium bicarbonate (SB) supplementation has been proposed as an ergogenic aid. Many studies indicate that oral SB intake increases blood pH and HCO_3_^-^ concentrations [11–13]. The increased H^+^ gradient on monocarboxylate transporters (MCTs) that carry lactate across muscle cell membranes, induced by SB ingestion augments the efflux of lactate and H^+^ from muscle cells to circulation [14]. It has also been suggested that metabolic alkalosis in skeletal muscles results in the acceleration of glycogenolysis, increasing the reliance on muscle glycogen stores as a fuel during exercise [15].

Nonetheless, the application of SB in athletic conditions demands considering the influence of aforementioned metabolic perturbations on exercise capacity and performance. High-intensity exercise has been shown to improve under SB in various studies [12, 16–19]. In a meta-analysis by Carr et al. [20], oral ingestion of SB at a dose of ~0.3 g∙kg^-1^ resulted in a moderate performance augmentation of 1.7% in a single 1-minute sprint with a further ~1% improvement in repeated sprint performance. Furthermore, in a hypertrophy-type exercise protocol (squat, leg press, and knee extension, utilizing four sets each, with 10–12 repetitions- of maximum loads and short rest periods between sets), SB increased the total number of completed repetitions [11]. Similarly, the ability to perform back squat repetition to failure was enhanced after SB ingestion [21]. Peart et al. [22] in the metaanalysis of 40 randomized clinical trials including 395 subjects also confirmed a favourable effect of sodium bicarbonate supplementation on physical performance. Authors of this meta-analysis concluded i.a. that an individual protocol of bicarbonate supplementation should be developed to avoid adverse effects and examination of NaHCO_3_ supplementation in athletes practicing technical disciplines can show its real effect on physical exercise.

CrossFit training consists of Olympic lifting, power lifting and gymnastic movements in varying combinations, loads, and repetition schemes with limited or no rest time between workout sets [23]. Thus, CrossFit training incorporates both, high-intensity and resistance exercises. In theory, athletes could benefit largely from the supplementation of SB. However, to date, there are no studies examining the influence of SB on performance in CrossFit.

Although the athletic performance benefits under SB are well documented, the major limitation to its application in athletic conditions may be the gastrointestinal (GI) side effects, i.e. nausea, vomiting, bloating, and cramping [24]. Athletes experiencing GI distress are less likely to improve under SB [25]. Several methods have been proposed to alleviate GI symptoms after SB ingestion, including co-ingestion with a meal containing carbohydrates [26], multiday ingestion [27] and splitting doses [28]. To address this problem, in the present study, we proposed a chronic, progressive-dose SB loading regimen. We aimed to examine the effects of SB loading on CrossFit-like performance and aerobic capacity as well as biochemical response to high-intensity exercise.

## Materials and methods

### Ethics statement

The study was approved by the local ethical committee (Bioethics Committee at Poznan University of Medical Sciences, Poznan, Poland. Decision no. 173/15 of 5 February 2015) and written informed consent was obtained from all participants before the study began (S1 and S2 Files). All procedures were conducted in accordance with the ethical standards of the 1964 Helsinki Declaration. The trial study protocol is presented in supporting S1 and S2 Files.

### Participants

**Fig 1:**
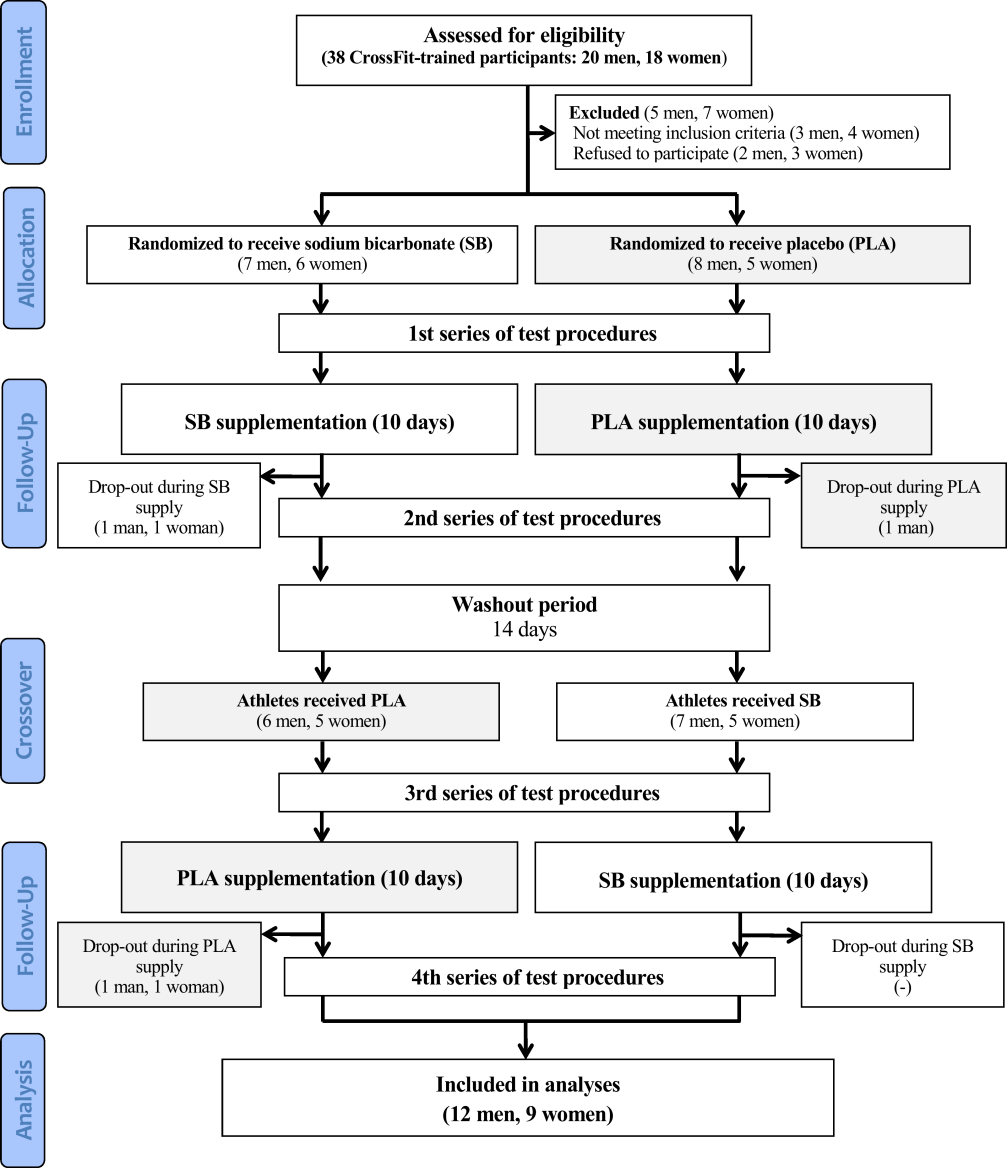
Flow diagram of the study design.

Twenty-six participants were initially enrolled in this study. However, 21 (9 women, 12 men; mean ± SD: age 32 ± 5 years, body height 174 ± 8 cm, baseline values of body mass 73.0 ± 14.0 kg_SB_ and 73.2 ± 13.8 kg_PLA_, fat free mass 58.4 ± 13.4 kg_SB_ and 59.3 ± 13.7 kg_PLA_, fat mass 13.6 ± 4.9 kg_SB_ and 13.9 ± 4.9 kg_PLA_) completed the entire study protocol and were included in analyses (Fig 1, S1 Table). The participants were recreationally and regularly training CrossFit at the RankorAthletics club in Poznań, Poland. The criteria for qualifying for the study, among others, included good health condition, a valid and up-to-date medical certificate confirming the athlete’s ability to practice sports, at least 2 years of CrossFit training experience, and a minimum of 4 workout sessions a week (minimum 3 in the practiced CrossFit discipline).

The studies were conducted from October 2015 to March 2016 at the Department of Human Nutrition and Hygiene (Poznań University of Life Sciences) and in RankorAthletics club in Poznań. All athletes declared that they had not introduced any changes in their lifestyles, elements of training, nutrition or supplementation, and that they had not been using any medications and supplements with potential ergogenic effects, other than those supplied by the authors of this study.

This trial was registered at Clinical Trials Gov (website: https://clinicaltrials.gov/ct2/show/NCT03165357; Clinical Trial Identification Number: NCT03165357). The study was registered retrospectively as registration was not required when the study enrolment started. The authors confirm that all ongoing and related trials for this intervention are registered. The study complies with the CONSORT Statement for randomized trials as shown in Fig 1 and S2 Table.

### Experimental protocol

The study protocol consisted of two 10-day experimental trials separated by a 14-day washout period in a randomized double-blind placebo-controlled cross-over manner (Fig 1). The participants were familiarized with the testing procedures, protocols, and equipment used before beginning the study. Anthropometric measures were obtained on the preliminary visit. After being qualified for the study, athletes were subjected to a randomization procedure (in a stratified design with lean body mass being a prognostic variable) and assigned either to the group receiving first a sodium bicarbonate preparation or to the group receiving placebo. The participants were first enrolled by the authors and then randomly assigned to the supplementation groups with specific codes by an impartial biostatistician.

The main study protocol involved another 4 visits to the laboratory. During trials, the participants ingested either SB or placebo (maltodextrin with NaCl). Pre and post each trial, the participants performed the incremental cycling test (ICT) and CrossFit-specific physical fitness test: Fight Gone Bad (FGB). All tests were performed at the same time each day. Blood samples were obtained pre and post each exercise test in order to measure blood lactate (La), pyruvate (Pa) and glucose concentrations, as well as creatine kinase (CK) and lactate dehydrogenase (LDH) activity. The participants were instructed to complete the anthropometric measurements and ICT testing sessions between 7.30 and 10.00 AM in a fasting state and avoid strenuous exercise for the 24 hours prior to each test session. However, three hours before the FGB tests, participants consumed standardized small meals.

In order to assess dietary intake, food diaries were given to the participants to record food and fluid consumption for the 3 days of each trial separately (before each laboratory visit). The participants also recorded training loads during both trials in training diaries. The participants were asked to maintain the same dietary intake and training load throughout the study protocol. In addition, the duration of washout period was established on the basis of a previous study [27], considering that it was even shorter (7 days) in other studies [18, 29]. The proper length of this period has also been established by studies reporting that one week was sufficient to remove any ergogenic effects of NaHCO_3_ supplementation [18, 29].

### Supplementation

In the SB trial, participants ingested SB in a progressive-dose regimen in order to reduce the likelihood of gastrointestinal side effects as presented in Table 1. On days 1–2, the dose of SB was: 37.5 mg∙kg^-1^ (25% of final dose 150 mg∙kg^-1^), on days 3–4: 75 mg∙kg^-1^ (50% of final dose), on days 5–7: 112.5 mg∙kg^-1^ (75% of final dose) and on days 8–10: 150 mg∙kg^-1^ (100% of final dose). SB was administered in the form of unmarked disk-shaped tablets (Alkala T, manufacturer -Sanum Kehbeck GmbH & Co. KG, Germany). The amount of SB in one tablet was 1 g. The tablets were ingested with at least 250 mL of water and could be either swallowed or dissolved in the mouth. In the placebo (PLA) trial, participants ingested maltodextrin (with NaCl) in a similar tablet form prepared by the same manufacturer as SB tablets. Both SB and PLA were ingested in 3 evenly split doses throughout the day. On training days, the supplements were taken in the morning, evening and 1.5 hours before each training session. On days of rest, the supplements were taken in the morning, afternoon and in the evening. The participants were also given individually adjusted supplementation schedules for each trial separately. For the purpose of double blinding, neither the researchers nor the participants knew whether SB or PLA was administered. Only the head of department had access to information on randomization, which was revealed only after cessation of the protocol.

**Table 1:** Supplementation protocol.

Day of trial	1	2	3	4	5	6	7	8	9	10
SB (mg ∙ kg^-1^)	37.5	37.5	75	75	112.5	112.5	112.5	150	150	150
PLA* (mg ∙ kg^-1^)	75	75	150	150	225	225	225	300	300	300

*–maltodextrin with NaCl

### Anthropometric measurements

At the preliminary visit to the laboratory, anthropometric measurements were taken in a fasting state in the morning hours. Body mass and height were measured using a professional medical scale with stadiometer (WPT 60/150 OW, RADWAG®, Poland) at an accuracy of 0.1 cm and 0.1 kg for height and body mass, respectively. Fat and free fat mass were assessed based on air displacement plethysmography using the Bod Pod® (Cosmed, Italy). Once the body density was determined, body fat and free fat mass were calculated using the Siri equation. Thoracic lung volume was estimated using the Bod Pod® software. During the measurement, participants wore only a swimsuit and an acrylic swim cap [30]. Total body water and hydration level was assessed by bioelectric impedance with Bodystat 1500 and by urine specific gravity measurements performed with a handheld refractometer; results <1.020 indicated proper hydratation. During these bioimpedance analyses, recommended measurement conditions were strictly followed [31].

### Exercise tests

Throughout the study protocol, the ICT and FGB workout were performed 4 times, pre and post each trial (SB or PLA). Prior to each test, participants were given instructions on the procedure and they completed a brief warm-up. All tests were performed in proper workout clothing and shoes, and the tests were supervised by an experienced researcher. Heart rate was continuously monitored during exercise using a telemetric system (Polar, Finland). Furthermore, capillary blood samples were obtained for analysis pre and post each test.

The ICTs were performed using the Kettler X1 cycloergometer (Germany) in the morning. The conditions at the laboratory were constant (temperature 20–22°C, relative humidity 60–70%). Each test started at a load of 50W for women and 75W for men. The load was then increased every 1.5 min by 25W. The point of fatigue was observed when a constant cadence of 65 ± 5 rpm could no longer be maintained despite further verbal encouragement provided by researchers. Throughout the exercise, inspired and expired gases were continuously analysed by a breath-by-breath system using the Quark CPET ergospirometer (Cosmed, Italy).

In order to assess CrossFit performance, the FGB workout was performed, which can also be used as a physical fitness test [32]. The FGB comprised of 5 multi-joint exercises (wall ball shots, sumo deadlift high-pulls, box jumps, push presses, and rowing) repeated in 3 rounds. Each FGB test was performed in the afternoon on the day after ICT. The participants were unstructured to perform as many repetitions of each exercise as they could for 1 minute, and then, immediately switched to the next exercise. Rounds were separated by one-minute breaks. The entire FGB test was video recorded to allow precise counting of all technically-well performed repetitions. Repetitions were accounted for, only if the participant completed a full range of motion required for each exercise. The whole FGB workout lasted for 17 min (3 rounds x 5 min and 2 breaks x 1 min; single 5-min round: 5 exercises x 1 min).

### Blood analysis

Fingertip blood samples were obtained immediately pre-exercise and 3 minutes post-exercise (ICT or FGB). All blood samples were obtained in a seated upright position, and immediately transferred to microtubes containing 500 μL of 0.6 M perchloric acid. CK activity was measured using an optimized kinetic method according to International Federation of Clinical Chemistry (IFCC) (Liquick Cor-CK, Cormay). LDH activity was measured using an optimized kinetic method of Deutsche Gesselschaft für Klinische Chemie (DGKC) (Liquick Cor-LDH, Cormay). Glucose concentration was measured using a colorimetric, enzymatic method with glucose oxidase (Liquick Cor-GLUCOSE, Cormay). [La] and [Pa] measurements were performed according to the method described previously by Maughan [33]. All biochemical analyses were conducted using a Synergy 2 SIAFRT microplate multi-detection reader (BioTek, USA).

### Gastrointestinal side effects

On days 1, 3, 5, 8 and 10 of supplementation, the participants completed an adapted questionnaire to measure gastrointestinal (GI) side effects [34]. The adapted questionnaire consisted of 19 items describing common GI symptoms. The numeric rating scale (NRS) (0–10 scale, with zero reflecting no GI distress at all and 10 being the most severe GI distress imaginable) was used to rate the intensity of these symptoms. It is important to mention that the questionnaire was not validated previously, which limits the interpretation of results. However, NRS is a valid and reliable tool recommended to measure patient-assessed global pain [35].

### Statistical analysis

The randomization was performed in a stratified design with lean body mass being a prognostic variable [36]. Standard methods for two-period two-treatment crossover designs were used to evaluate the effect of supplementation [37]. Next, the repeated measurements (pre and post) were analysed using the two-period two-treatment crossover design that incorporates R-side covariation at two levels to compare pre and post supplementation. The structure of covariation matrix was chosen based on the AIC criterion. The following effects were analysed: period, sequence, subject (participant) within sequence, treatment, time (pre and post supplementation) within period, and treatment x time interaction. The subject (participant) within sequence was a random effect, while all other effects were fixed.

More complex repeated measurements analysis using the two-period two-treatment crossover design, in which the cycles were accounted for, was performed for FGB and blood. The R-side covariation at two levels was also incorporated. The structure of covariation matrix was chosen based on the AIC criterion. In the analysis of FGB test, which consisted of 3 rounds, those rounds were treated as cycles, whereas in the analysis of blood samples, which were taken three times during one testing session (before exercise, after FGB and after ICT), those measurements were treated as cycles. Next, the following effects were analysed: period, subject (participant) within sequence, treatment, time (pre and post) within period, treatment x time interaction, cycle within treatment x time interaction [38]. The subject (participant) within sequence was a random effect, while all other effects were fixed. If significant effects were found, the Bonferroni post-hoc test was calculated to test the specific differences between averages (SB_pre_ : SB_post_, PLA_pre_ : PLA_post_ and SB_post_ : PLA_post_).

Statistical significance was set at p<0.05. The assumptions of normality and homoscedasticity were tested using the Shapiro-Wilk test for normality. If data did not meet the assumptions, the Box-Cox transformation was used. Data were analysed by manual calculations and using the STATISTICA 12 (StatSoft Inc., USA) and SAS 9.4 (SAS Institute Inc., USA) software programs. In order to achieve a 0.95 power to detect a 7.9% difference in the mean change of total number of all repetitions in FGB with α 0.05 using crossover ANOVA, a total sample size of 18 would be required. However, repeated ANOVA required 12 individuals to achieve a 0.95 power with α 0.05 for two groups and four repeated measurements. Mean correlation between repeated measures and medium effect size were obtained from our pilot study. The correlation between repeated measures was 0.85 and partial eta^2^ = 0.06. Power calculations were performed in G*Power 3 (Heinrich-Heine-University Düsseldorf, Germany) software program.

## Results

There were no significant sequence effects for any of the response variables (p>0.05). No carryover effects were observed, demonstrating that the 14-day washout period was sufficient.

### Gastrointestinal side effects

Progressive-dose supplementation regimen was well tolerated. None of the participants reported any meaningful gastrointestinal side effects (score >5 on a numeric rating scale) throughout the study protocol (S3 Table).

### Performance in FGB test

**Fig 2:**
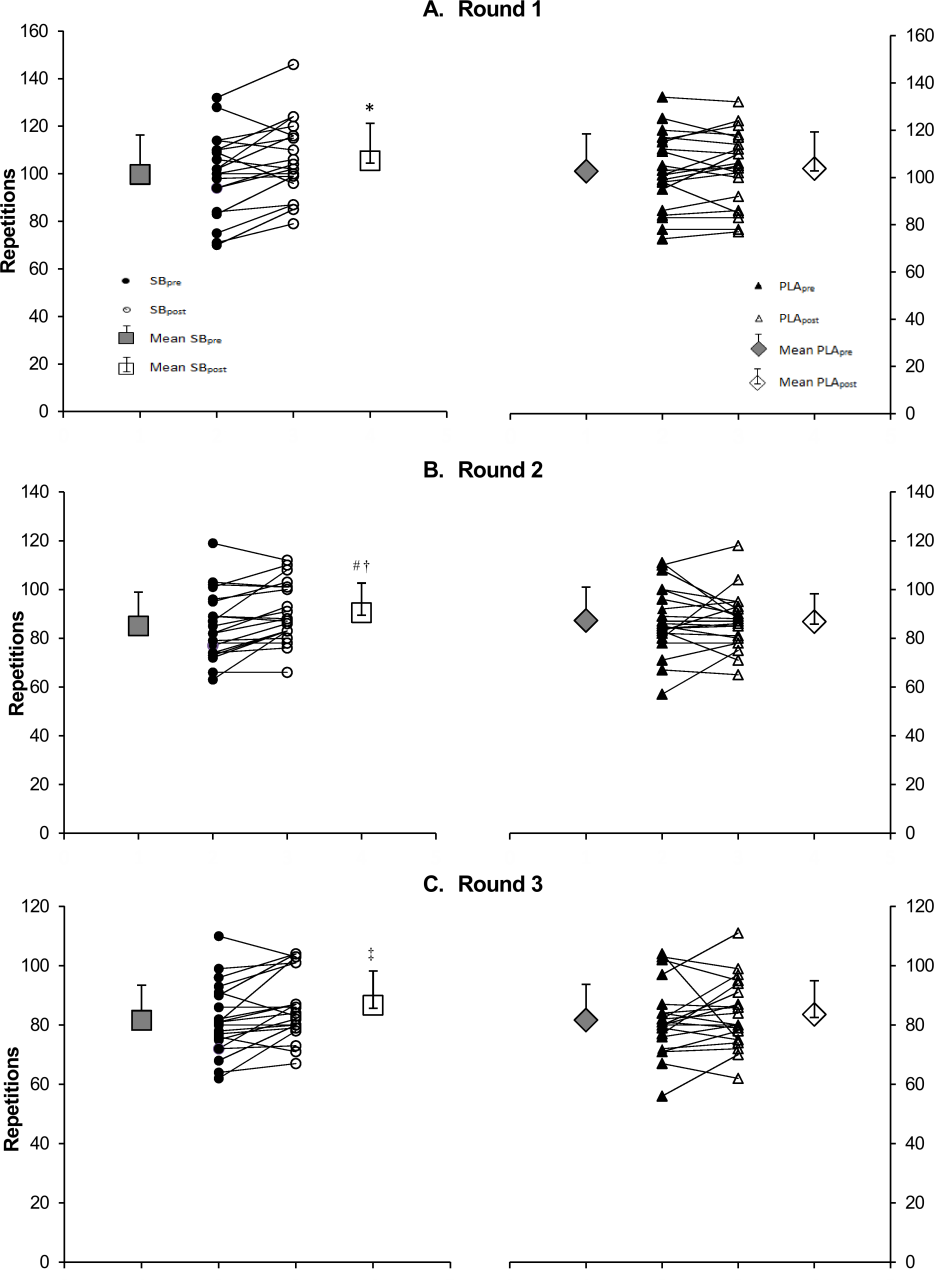
Performance in each Fight Gone Bad round. Panel A—repetitions completed in round 1, panel B—repetitions completed in round 2 and panel C—repetitions completed in round 3. SB_pre_, before sodium bicarbonate trial; SB_post_, after sodium bicarbonate trial; PLA_pre_, before placebo trial; PLA_post_, after placebo trial. Significantly different from SB_pre_: * p = 0.002 (round 1), ^#^ p = 0.0002 (round 2), ^‡^ p = 0.021 (round 3), significantly different from PLA_post_: ^†^ p = 0.036 (round 2).

**Fig 3:**
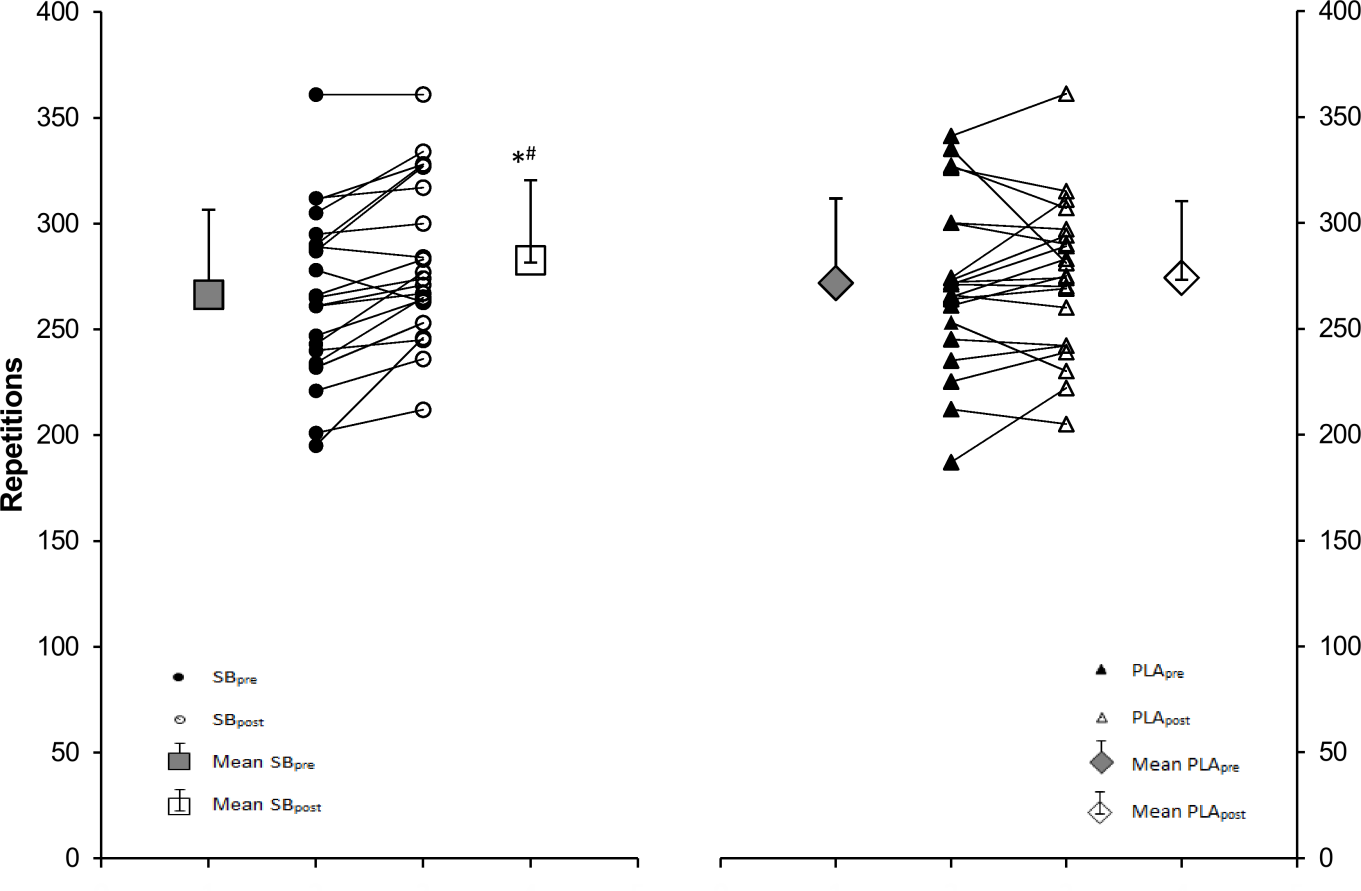
Total number of repetitions in all rounds of Fight Gone Bad test. SB_pre_, before sodium bicarbonate trial; SB_post_, after sodium bicarbonate trial; PLA_pre_, before placebo trial; PLA_post_, after placebo trial. Significantly different from SB_pre_: * p<0.001; significantly different from PLA_post_: ^#^ p = 0.040.

Exercise intensity was monitored continuously during FGB test (S4 Table). The number of correctly performed repetitions in FGB test increased in round 1 under SB from 99.7 ± 16.6 reps SB_pre_ to 105.5 ± 15.7 reps SB_post_, p = 0.002 (~5.8% change), in round 2 from 85.0 ± 13.9 reps SB_pre_ to 90.5 ± 12.2 reps SB_post_, p = 0.0002 (~6.4% change) and in round 3 from 81.6 ± 11.8 reps SB_pre_ to 86.7 ± 11.6 reps SB_post_, p = 0.021 (~6.2% change) (Fig 2). In round 2, the number of completed repetitions was significantly higher SB_post_ than PLA_post_ (90.5 ± 12.2 reps SB_post_ vs. 86.8 ± 11.4 reps PLA_post_, p = 0.036). However, no significant differences were observed following placebo. The total number of repetitions of all exercises in all 3 rounds was 3.1% higher SB_post_ than PLA_post_ [282.6 ± 37.9 reps vs. 274.1 ± 36.2 reps, respectively (p = 0.040)] (Fig 3). In addition, a 6.1% improvement in total number of repetitions of all exercises in FGB following SB loading was observed (266.4 ± 40.2 reps SB_pre_ vs. 282.6 ± 37.9 reps SB_post_, p<0.001) (Fig 3).

Furthermore, the repeated measures analysis of variance, which included the two-period two-treatment crossover design, indicated a significant effect of round (time x treatment) (p<0.0001). Further, the Bonferroni test showed significant differences between all pairs of rounds within time x treatment. However, the number of repetitions between SB_pre_ and SB_post_ improved only in round 1 (p<0.0001). Moreover, an improvement in the number of repetitions between SB_pre_ and SB_post_ for the aforementioned variables (SB_pre_ vs. SB_post_: total number of repetitions done in each round, p = 0.0032) was observed.

### Performance in ICT

**Fig 4:**
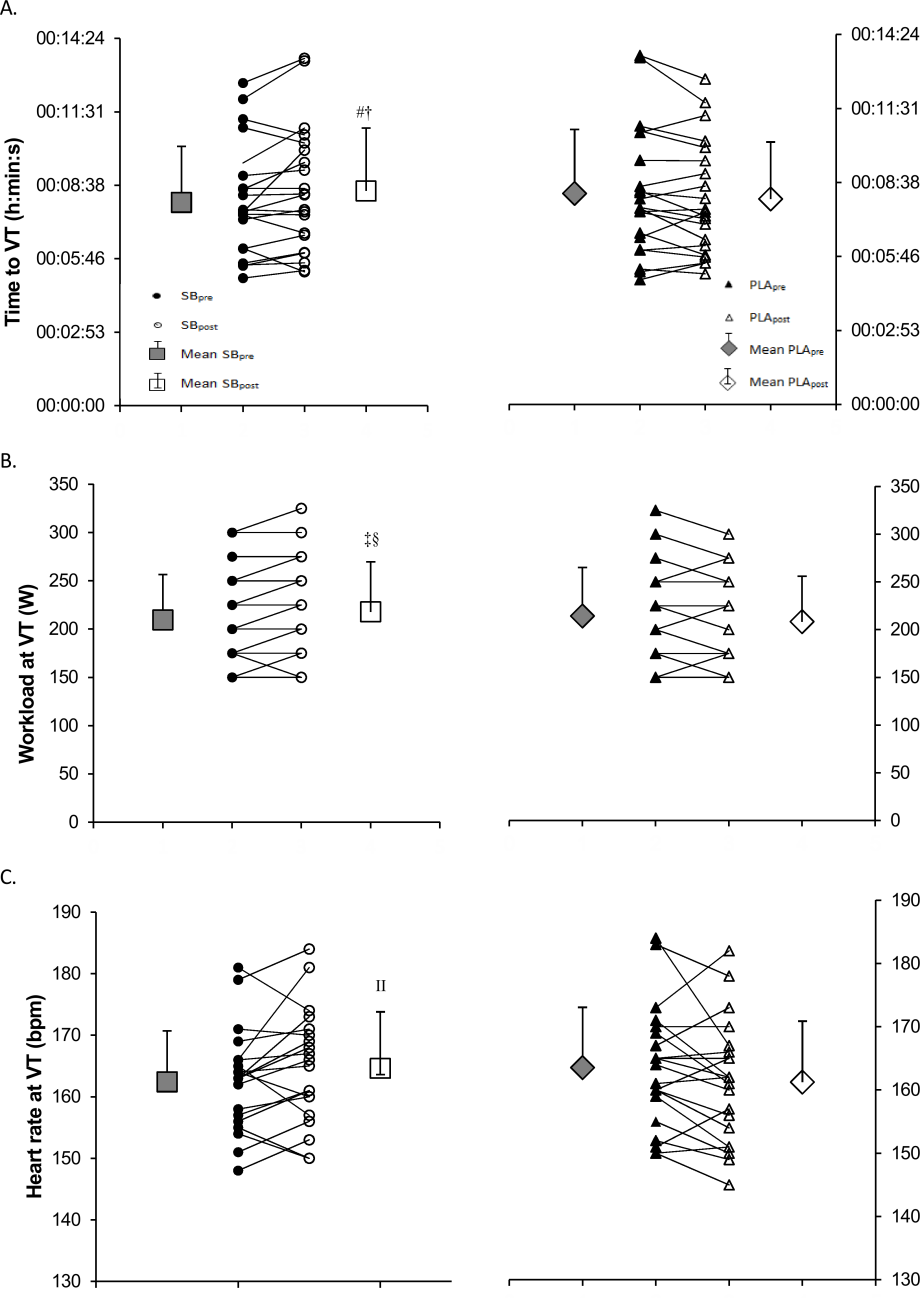
Ventilatory threshold in incremental cycling test. Panel A—time to VT, panel B -workload at VT and panel C—heart rate at VT. Significantly different from SB_pre_: ^#^ p = 0.027 (time to VT), ^‡^ p = 0.028 (workload at VT); significantly different from PLA_post_: ^†^ p = 0.020 (time to VT), ^§^ p = 0.014 (workload at VT), ^II^ p = 0.020 (heart rate at VT).

Time to exhaustion, maximum workload and maximum HR in the ICT were not significantly different after SB supplementation (Table 2). However, workload at VT increased from 210 ± 47 W SB_pre_ to 218 ± 52 W SB_post_, p = 0.028 (~4.0% change) after SB supplementation (Table 2), and it was 4.6% higher at SB_post_ than at PLA_post_ (218 ± 52 W SB_post_ vs. 208 ± 48 W PLA_post_, p = 0.037), respectively. Time to VT increased from 7:58 ± 2:12 min SB_pre_ to 8:25 ± 2:28 min SB_post_, p = 0.027 (~4.2% change) after SB supplementation and it was ~5.1% higher at SB_post_ than at PLA_post_ (8:25 ± 2:28 min SB_post_ vs. 8:00 ± 2:13 min PLA_post_, p = 0.020), respectively (Fig 4). However, the heart rate at VT was significantly higher at SB_post_ than at PLA_post_ (165 ± 9 bpm SB_post_ vs. 161 ± 10 bpm PLA_post_, p = 0.030). Furthermore, the maximum carbon dioxide production was elevated after SB loading from 3604 ± 955 ml∙min^-1^ SB_pre_ to 3776 ± 1020 ml∙min^-1^ SB_post_, p = 0.049 (~4.8% change).

**Table 2:** Performance and ventilatory threshold in incremental cycling test (ICT).

	SB_pre_	SB_post_	PLA_pre_	PLA_post_
**T**_**exh**_ **(min:s)**	10:54 ± 3:26	11:12 ± 3:16	10:56 ± 3:17	11:14 ± 3:44
**W**_**max**_ **(W)**	260 ± 63	262 ± 63	260 ± 63	268 ± 72
**HR**_**max**_ **(bpm)**	175 ± 7	177 ± 8	176 ± 75	176 ± 9
**VO**_**2**_**max (mL∙min**^**-1**^**)**	3230 ± 808	3276 ± 917	3273 ± 910	3241 ± 893
**VCO**_**2**_**max (mL∙min**^**-1**^**)**	3604 ± 955	3776 ± 1020*	3672 ± 962	3716 ± 1036
**T**_**VT**_ **(min:s)**	7:58 ± 2:12	8:25 ± 2:28^#^^†^	8:13 ± 2:29	8:00 ± 2:13
**W**_**VT**_ **(W)**	210 ± 47	218 ± 52 ^‡^^§^	214 ± 51	208 ± 48
**HR**_**VT**_ **(bpm)**	162 ± 8	165 ± 9 ^II^	164 ± 10	161 ± 10
**VE**_**max**_ **(L∙min**^**-1**^**)**	121 ± 35	125 ± 36	122 ± 36	124 ± 37

Data are mean ± SD. T_exh_, time to exhaustion; W_max_, maximum workload; HR_max_, maximum heart rate; VO_2_max, maximum oxygen uptake; VCO_2_max, maximum carbon dioxide production; T_VT_, time to ventilatory threshold (VT); W_VT_, workload at VT; HR_VT_, heart rate at VT; VE_max_, maximal minute ventilation. Significantly different from SB_pre_

* p = 0.049

^#^ p = 0.027

^‡^ p = 0.028; significantly different from PLA_post_

^†^ p = 0.020

^§^ p = 0.037

^II^ p = 0.030.

### Blood sample analysis

Blood [La], [Pa] and [glucose] concentrations, as well as LDH and CK activity increased after both exercise tests regardless of the experimental trial (Table 3). Statistically significant differences between SB and PLA trials were only observed in [Pa] and CK activity at rest before the ICT. Before the onset of ICT, pre exercise [Pa] increased from 0.25 ± 0.06 mmol∙L^-1^ SB_pre_ to 0.32 ± 0.11 mmol∙L^-1^ SB_post_ (p = 0.001) and was significantly higher compared to that after placebo treatment (0.32 ± 0.11 mmol∙L^-1^ SB_post_ vs. 0.26 ± 0.09 mmol∙L^-1^ PLA_post_). Furthermore, CK activity before the onset of ICT decreased after both trials (SB and PLA).

**Table 3:** Biochemical response to exercise tests (Fight Gone Bad and incremental cycling test).

		SB_pre_	SB_post_	PLA_pre_	PLA_post_
**[La]** (mmol∙L^-1^)	**FGB**_**pre**_	1.75 ± 1.02	1.51 ± 0.79	1.54 ± 0.78	1.42 ± 0.54
	**FGB**_**post**_	11.23 ± 2.45*	11.77 ± 2.56*	11.07 ± 2.85*	10.91 ± 3.39*
	**ICT**_**pre**_	1.05 ± 0.45	1.18 ± 0.77	1.12 ± 0.59	1.15 ± 0.45
	**ICT**_**post**_	10.79 ± 3.12*	10.91 ± 3.29*	10.63 ± 2.64*	11.21 ± 2.50*
**[Pa]** (mmol∙L^-1^)	**FGB**_**pre**_	0.32 ± 0.08	0.31 ± 0.08	0.32 ± 0.08	0.28 ± 0.07
	**FGB**_**post**_	0.66 ± 0.12*	0.69 ± 0.11*	0.67 ± 0.11*	0.62 ± 0.13*
	**ICT**_**pre**_	0.25 ± 0.06	0.32 ± 0.11^#^^†^	0.30 ± 0.08	0.26 ± 0.09^‡^
	**ICT**_**post**_	0.66 ± 0.12*	0.66 ± 0.15*	0.65 ± 0.09*	0.66 ± 0.11*
**[Glucose]** (mg∙dL^-1^)	**FGB**_**pre**_	99.1 ± 18.9	93.0 ± 10.7	94.7 ± 11.4	94.9 ± 19.2
	**FGB**_**post**_	127.5 ± 33.0*	126.0 ± 31.8*	123.9 ± 32.5*	123.8 ± 29.9*
	**ICT**_**pre**_	87.1 ± 10.1	90.1 ± 17.8	85.8 ± 9.2	93.8 ± 17.0^§^
	**ICT**_**post**_	114.6 ± 29.8*	109.3 ± 19.3*	106.8 ± 23.4*	108.3 ± 21.2*
**LDH** (U∙L^-1^)	**FGB**_**pre**_	280 ± 64	293 ± 82	288 ± 84	271 ± 90
	**FGB**_**post**_	316 ± 80*	326 ± 84*	316 ± 76*	324 ± 90*
	**ICT**_**pre**_	299 ± 92	286 ± 63	286 ± 102	272 ± 79
	**ICT**_**post**_	332 ± 91*	345 ± 84*	318 ± 87*	352 ± 76*
**CK** (U∙L^-1^)	**FGB**_**pre**_	249 ± 83	246 ± 72	254 ± 95	243 ± 73
	**FGB**_**post**_	274 ± 91*	277 ± 99*	283 ± 112*	283 ± 92*
	**ICT**_**pre**_	287 ± 137	275 ± 101^II^	305 ± 140	250 ± 110^††^
	**ICT**_**post**_	301 ± 136*	310 ± 112*	323 ± 144*	293 ± 121*

Data are mean ± SD. FGB, Fight Gone Bad; ICT, incremental cycling test; La, lactate; Pa, pyruvate; LDH, lactate dehydrogenase; CK, creatine kinase.

* Significantly different from pre exercise (pre FGB or pre ICT): p<0.05

^#^ significantly different from SB_pre_: p = 0.001

^†^ significantly different from PLA_post_: p = 0.001

^‡^ significantly different from PLA_pre_: p = 0.017

^§^ significantly different from PLA_pre_: p = 0.009

^II^ significantly different from PLA_post_: p = 0.010

^††^ significantly different from PLApre: p = 0.024.

## Discussion

The current study is the first to assess the effects of sodium bicarbonate supplementation on CrossFit-like performance. We observed that chronic intake of sodium bicarbonate in a progressive-dose loading regimen did not elicit any meaningful gastrointestinal side effects and concomitantly improved CrossFit-like performance. In addition, the ingestion of sodium bicarbonate delayed the occurrence of ventilatory threshold, as well as the workload and heart rate at VT in the incremental cycling test. However, it was not accompanied by any further alterations in blood [La] after the exercise tests. Only resting [Pa] was significantly higher following sodium bicarbonate supplementation before the incremental cycling test, but it was still within the range of reference values. Nevertheless, it is possible that the duration of ICT test was too short, for post exercise [La] and [Pa] changes in blood to be observed after SB supplementation. Following SB supplementation, the resting CK activity before ICT decreased indicating better adaptation. Furthermore, there was no effect of sodium bicarbonate loading on blood [glucose], and CK or LDH activity after both exercise tests (FGB and ICT).

From a practical point of view, exercise improvements observed after SB ingestion should translate into greater training adaptation. However, a factor limiting its application is the gastrointestinal side effects [24]. To overcome this issue, in the present study, we proposed a progressive-dose 10-day loading regimen. The highest doses of SB were administered from day 8 to 10 amounting to 150 mg∙kg^-1^. Although they were 2X lower than the doses previously advised [11], they elicited performance improvements. In addition, none of 21 participants in the present study reported any meaningful gastrointestinal side effects, suggesting a good tolerance. This is particularly important, because in a previous study, the administration of higher doses (300 mg∙kg^-1^) resulted in severe GI distress [24]. GI symptoms can be caused by CO_2_ released due to a reaction in the stomach, where NaHCO_3_ rapidly reacts with hydrochloric acid to form NaCl, CO_2_, and H_2_O [39]. The hypertonic sodium bicarbonate solutions may cause an intraluminal osmotic load and water shift from plasma and extracellular fluid to the intestinal lumen [40]. In a study by Kahle et al. [24], global ratings of gastrointestinal distress severity were greater after SB ingestion and the most frequent symptoms included diarrhoea, bloating, thirst, and nausea.

Saunders et al. [25] showed that only when participants experiencing GI discomfort were excluded from analysis, exercise capacity improvement was evident. Hence, different SB supplementation regimens have been proposed in the literature to alleviate GI discomfort [26–28]. Previously, splitting doses reduced the incidence of GI distress, with only 3 out of 20 participants reporting severe GI symptoms during high-intensity cycling efforts [25]. Co-ingestion with a small high-carbohydrate meal is another effective strategy to prevent GI distress after administration of SB [28]. Furthermore, it was advised that a dose of 300 mg∙kg^-1^ should be taken 120–150 min before the exercise [26]. However, the GI symptoms could be eliminated only when the dose was reduced to a maximum of 150 mg∙kg^-1^ based on a previous adaptation to smaller doses, as in the present study. Despite this, the questionnaire used in our study to measure gastrointestinal (GI) side effects was not previously validated, which limits the interpretation. The tolerance and compliance of patients using NaHCO_3_ also depends on the galenic form of the product [39]. The product used in our study is registered in Germany and was produced according to the pharmaceutical requirements and general medical practitioner conditions.

In the present study, the overall CrossFit-like performance improved by ~6% following SB supplementation. Total number of repetitions of all exercises in each round also increased after SB administration. These results are in accordance with a previous study by Carr et al. [11], in which acute SB ingestion increased the total number of repetitions completed throughout the resistance exercise protocol. However, the numbers of back squat, inclined leg press and knee extension repetitions analysed separately were not significantly different following SB in that study. In an exercise-to-failure protocol [21], the acute ingestion of SB increased only the number of repetitions of the first exercise (back squat) compared to placebo, with no effect on the second exercise (bench press). Therefore, it can be suggested that the ergogenic effects of SB and training adaptations are more evident with chronic rather than acute SB supplementation, as proposed in the current study. It is also worth-mentioning that dose used in the current study were smaller (ranging from 37.5 to 150 mg∙kg^-1^) compared to that (300 mg∙kg^-1^) administered in the aforementioned studies [11,21]. It is therefore possible that increasing the SB dose up to 300 mg∙kg^-1^, using progressive-dose ingestion could further improve the level of athlete’s physical capacity.

Similar to those of the resistance exercise, results of the influence of SB supplementation on high-intensity intermittent exercise were equivocal. In a study by Krustrup et al. [19], the Yo-Yo intermittent recovery test performance was augmented by 14% when SB (400 mg∙kg^-1^) was ingested. Furthermore, rating of perceived exertion was lowered, and blood alkalosis was elevated. In a repeated sprint test [29], the intake of 300 mg∙kg^-1^ of SB resulted in a higher total amount of work completed during the test through enhanced buffering capacity. However, 4-week SB supplementation combined with high-intensity training failed to further improve performance [12]. These discrepancies might be due to differences in the athletic status of participants, different exercise protocols, GI side effects and the duration of supplementation. The performance improvements elicited by SB ingestion are considered to be related to the enhanced buffering ability [11–13, 39] and a concomitant increase in the rate of H^+^ removal from working muscles. The maintenance of intramuscular pH allows maintenance of muscle contractility, thus delaying the onset of fatigue [8].

In the present study, VT was delayed by ~27 s following SB supplementation and occurred at higher workloads (~4%) and heart rates (~2%) during the incremental cycling test. The maximum production of carbon dioxide was ~5% elevated, but no difference was observed in the highest oxygen uptake. However, these alterations had no influence on the capacity to perform the incremental cycling test, with no differences in time to exhaustion, maximum workload, or maximum heart rate. The shift in VT was not accompanied by significant alterations in blood [La], but it has been well-established that VT is not dependent on lactate production [41]. Similarly, in a study on cyclists [42], no performance improvements in ICT were observed following the acute ingestion of SB. In contrast to our results, the maximum oxygen uptake during ICT slightly decreased under SB in that study, which was explained by the left shift of the oxyhaemoglobin dissociation curve during alkalosis, evidenced by lowered partial pressure of oxygen under SB and unaltered oxygen saturation [42].

It is also worth mentioning that in a study by Edge et al. [43], SB supplementation combined with interval training for 8 weeks improved lactate threshold (LT), but had no impact on the VO_2_max [41]. Since VO_2_max seems to be more reliant on oxygen delivery than utilization in the muscle cells [44], the improvement observed in LT was hypothesized to result from the enhanced oxidative capacity within the muscles, which was connected to improved mitochondrial adaptation [43]. Indeed, more recent research has shown that acute ingestion of SB prior to high-intensity intermittent exercise enhances the expression of PGC-1α mRNA during recovery, suggesting a potential mechanism for improved mitochondrial adaptation [13]. Therefore, in the current study, VO_2_max remained unaltered but VT improved after SB administration.

A related point to consider is that increased CO_2_ production during anaerobic exercise results from higher rates of H^+^ production and release from muscle cells into the blood, where H^+^ ions are neutralized by, bicarbonate (HCO_3_^-^) among others, as expressed by the equation: H^+^ + HCO_3_^-^ = H_2_CO_3_ = H_2_O + CO_2_ [45]. The increase in buffering capacity after SB ingestion could potentially influence the production of CO_2_, when the H^+^ efflux is enhanced [46]. Higher VCO_2_max after SB ingestion, as reported in the current study, was also observed during short supramaximal exercises [47].

In the present study, despite the increase in VCO_2_max, no further elevation in blood [La] under SB after the cessation of exercise tests was reported. Nor was the [Pa], [glucose] and CK and LDH activity different between trials. Following 10 days of SB supplementation, [Pa] and CK activity at rest were elevated only prior to beginning the incremental cycling test blood, compared to placebo. However, after cessation of ICT, blood [La] and [Pa] levels under SB were not different from those after the placebo trial or from SB_pre_. These results are in contrast to those of previous studies, in which blood [La] was further increased post-exercise under SB compared to placebo [11, 19, 42–43]. We suspect that the doses used in the current study might be too small to elicit lactate efflux to the extent observed in previous studies. The fact that time to exhaustion in the present study increased non-significantly under both SB and placebo is also worth discussing. Participants were well familiarized with the ICT protocol prior to beginning the study. However, a practice effect might have influenced the results, even though they were not statistically significant. Another limitation of our study is the small sample size. Perhaps more observations would have been statistically significant with a larger sample size. Moreover, because the sample size was drawn from a fairly specific population, the generalizability of study results might be limited.

## Conclusions

This study indicated that progressive-dose chronic sodium bicarbonate supplementation eliminated the of incidence gastrointestinal discomfort observed with acute dosing. Even though the dosage seemed to be too small to elicit further lactate responses in the blood as previously reported, significant improvements in CrossFit-like performance were observed, with potential for further enhancement on higher doses. In addition, the heart rate and workload at ventilatory threshold were augmented and the occurrence of ventilatory threshold was delayed after SB ingestion, suggesting better metabolic adaptation. Nonetheless, further studies on the effects of chronic SB ingestion are necessary to fully understand the underlying mechanisms contributing to training adaptations in CrossFit-like exercise.

## Supporting information

S1 File Original study protocol approved by our IRB. (PDF)

S2 File Study protocol approved by our IRB. (PDF)

S1 Table Baseline characteristics of the participating athletes. (PDF)

S2 Table CONSORT checklist. (PDF)

S3 Table Gastrointestinal side effects during SB and PLA supplementation. (PDF)

S4 Table Heart rates in Fight Gone Bad test. (PDF)
