# Supplementary material for: The effect of chronic progressive-dose sodium bicarbonate ingestion on CrossFit-like performance: A double-blind, randomized cross-over trial
Source: PLoS One. 2018 May 17;13(5):e0197480. doi: 10.1371/journal.pone.0197480 (PMC5957406; doi:10.1371/journal.pone.0197480)
Supplement: S1 File — (PDF) [file pone.0197480.s001.pdf]

Uniwersytet Przyrodniczy w Poznaniu
Wydział Nauk o Żywności i Żywieniu
Katedra Higieny Żywienia Człowieka
Kierownik Katedry: Prof. dr hab. Jan Jeszka
Kierownik Badań: Krzysztof Durkalec-Michalski
Wojska Polskiego 31, 60-637 Poznan, Poland
Phone/Fax: (+48 61) 848 73 32

PROTOKÓŁ BADAŃ

WPŁYW SUPLEMENTACJI DWUWĘGLANEM SODU W WYBRANEJ GRUPIE SPORTOWCÓW

1. Wprowadzenie

Wysiłek fizyczny o wysokiej intenysywności w warunkach niedoboru tlenu powoduje produkcję kwasu mlekowego na drodze glikolizy beztlenowej (Coso i wsp., 2010). Kwas mlekowy ulega natomiast hydrolizie do mleczanu i jonów wodorowych, co powoduje obniżenie pH. Nasilające się zakwaszenie mięśni jest czynnikiem, który wpływa na wystąpienie uczucia zmęczenia (Siegler i wsp., 2016). Dlatego tak istotne jest prawidłowe funkcjonowanie mechanizmów buforujących, do których zaliczają się dwuwęglany krwi. Suplementacja dwuwęglanem sodu zwiększa pojemność buforową krwi, dzięki czemu może wpływać także na zdolność do wykonywania wysiłku fizycznego. Meta-analiza dostępnych badań wykazała, że suplementacja dwuwęglanem sodu powoduje 1,7-procentową poprawę wyniku w 1-minutowym sprincie, a przy powtarzanych sprintach poprawa wyniku była nawet o 1 % większa (Carr i wsp., 2011). W kolejnej meta-analizie również wykazano korzystny wpływ suplementacji dwuwęglanem sodu na wynik sportowy i podkreślono, że niezbędne jest opracowanie protokołu supementacyjnego, który zapobiegałby także wystąpieniu efetów ubocznych ze strony układu pokarmowego, które u wielu sportowców stanowią przeciwwskazanie do stosowania suplementacji dwuwęglanem sodu (Peart i wsp., 2012). Ponieważ CrossFit należy do sportów o wysokiej intensywności wysiłku, wydaje się, że suplementacja dwuwęglanem sodu w grupie jego adeptów mogłaby mieć potencjalnie

korzystny wpływ na zdolności wysiłkowe. Jednak do tej pory takie badania nie zostały przeprowadzone.

2. Specyfika prowadzonej suplementacji

Badania krzyżowe, randomizowane z podwójnie ślepą próbą.

Grupa przyjmująca dwuwęglan sodu: progresywna suplementacja trwająca 10 dni, dawka w pierwszym dniu suplementacji będzie wynosiła 37,5 mg $\cdot$ kg^{-1}, a w ostatnim dniu 150 mg $\cdot$ kg^{-1}. Dawkowanie zostało ustalone w celu ograniczenia niekorzystnych efektów ubocznych wywołanych przyjmowaniem większych dawek suplementu (Kahlea i wsp., 2013). Dwuwęglan sodu będzie podawany w formie tabletek do ssania w kształcie dysku (Alkala T, manufacturer - Sanum Kehbeck GmbH & Co. KG, Germany). Zawartość dwuwęglanu sodu w 1 tabletce będzie wynosiła 1 g. Spożywane będą w trzech porcjach dziennie z min. 250 ml wody. W dni treningowe suplementy będą przyjmowane rano, wieczorem i 1,5 h przed treningiem. W dni beztreningowe: rano, popołudniu i wieczorem. Pomiędzy etapem suplementacji a placebo uczestnicy będą mieli 14-dniową przerwę, tzw. *washout*.

Grupa przyjmująca placebo: placebo stanowić będzie preparat maltodekstryny (z dodatkiem NaCl) w podobnej formie dyskowatej tabletki. Tabletki spożywane będą w trzech porcjach dziennie z dodatkiem min. 250 ml wody. W dni treningowe pacebo przyjmowane będą rano, wieczorem i 1,5 h przed treningiem. W dni beztreningowe: rano, popołudniu i wieczorem. Pomiędzy etapem placebo a suplementacji uczestnicy będą mieli 14-dniową przerwę, tzw. *washout*.

3. Charakterystyka badanej populacji

W badaniach udział weźmie około 30 osób (mężczyźni i kobiety), w wieku 20-43 lat. Uczestnicy będą stanowili grupę osób trenujących CrossFit. Warunki włączenia będą obejmowały dobry stan zdrowia, aktualne badania lekarskie zezwalające na uprawianie sportów, conajmniej dwuletnie doświadczenie w trenowaniu CrossFit i miminum 4 jednostki treningowe w tygodniu (w tym conajmniej 3 z zakresu CrossFit).

4. Metody badań

4.1. Ocena wskaźników antropometrycznych i składu ciała

4.1.1. Ocena masy i wysokości ciała

Ocena masy i wysokości ciała badanej grupy zawodników zostanie wykonana przy użyciu, posiadającej certyfikat zgodności z normą i dyrektywą medyczną, legalizowanej wagi lekarskiej, połączonej z antropometrem typu WPT 60/150 OW firmy RADWAG® (Polska). Analiza masy i wysokości ciała będzie odbywała się w godzinach porannych z zastosowaniem wszelkich rekomendowanych procedur pomiarowych (CDC 2007).

4.1.2. Ocena składu ciała metodą bioimpedancji elektrycznej

Analiza składu ciała metodą bioimpedancji elektrycznej zostanie wykonana z wykorzystaniem czteroelektrodowego analizatora BIA 101S AKERN-RJL (Włochy) i oprogramowania Bodygram 1.31 (Włochy).

Analiza składu ciała zostanie wykonana z zachowanie wszelkich rekomendowanych warunków pomiarowych m.in. w godzinach porannych, w stanie na czczo, w pozycji leżącej i z zastosowaniem właściwej metodyki aplikacji elektrod pomiarowych (Kyle i wsp., 2004; Lukaski i Johnson 1986).

Sportowcy być również zobowiązani do powstrzymania się od picia kawy, herbaty, produktów zawierających kofeinę i alkohol, przez co najmniej 24 godzin przed testem, jak i powstrzymania się od wykonywania wysiłku fizycznego, przez co najmniej 18 godzin przed pomiarami.

4.1.3. Ocena składu ciała metodą pletyzmografii

Ocena składu ciała metodą pletyzmografii zostanie wykonana z wykorzystaniem analizatora Bod Pod (Cosmed srl, Włochy). Analiza gęstości ciała na podstawie pletyzmograficznej, wielopunktowej oceny wyporności powietrza umożliwia precyzyjną analizę komponentów składu ciała (m.in. zawartości tłuszczu, beztłuszczowej masy ciała). Badania składu ciała metodą pletyzmografii zostaną wykonane z zachowaniem wszystkich procedur i zaleceń

dotyczących metodyki przeprowadzenia tego typu badań (Bentzur i wsp. 2008). Uczestnicy podczas badania będą ubrani jedynie w strój kąpielowy oraz czepek pływacki. W badaniach określona zostanie także objętość gazu, zgromadzonego w drogach oddechowych. Wyniki pomiarów przeliczone będą z wykorzystaniem równania Siri, przy użyciu oprogramowania, załączonego do analizatora Bod Pod.

4.2. Specyficzna próba wysiłkowa *„Fight Gone Bad”*

„Fight Gone Bad” jest specyficzną próbą służącą do określenia wydolności fizycznej osób uprawiających CrossFit, która może jednocześnie stanowić samodzielną jednostkę treningową (Goins, 2014). Każdy badany będzie czterokrotnie brał udział w treningu poprzedzonym rozgrzewką, zawsze o tej samej porze, w godzinach popołudniowych. Podczas próby będzie monitorowano częstość skurczów serca za pomocą pulsometrów Polar RS400 (Finlandia) wraz z paskiem z nadajnikiem umieszczanym na klatce piersiowej. Cały trening będzie również nagrywany za pomocą kamery. Obraz uzyskany dzięki nagraniu pozwoli na indywidualne obliczenie wszystkim uczestnikom powtórzeń wykonanych podczas treningu. Jednostka treningowa FGB będzie polegać na wykonaniu jak największej ilości powtórzeń każdego z 5 ćwiczeń w ciągu 1 minuty. Seria pięciu ćwiczeń trwać będzie 5 minut, a cały trening będzie się składać z 3 serii, między którymi będą występowały 1- minutowe przerwy (łączny czas trwania wyniesie 17 min). Wysiłki wykonywane podczas treningu FGB będą obejmowały:

- *sumo deadlift high- pulls (sumo)* - podnoszenie sztangi na wysokość barków, sztanga umieszczona na ziemi, ćwiczący w lekkim rozkroku pochyla się po sztangę, utrzymując proste plecy, przy każdym powtórzeniu należało odłożyć sztangę do pozycji wyjściowej;
- *wall ball shots (wall ball)* - rzuty piłką lekarską o masie 9 kg do tarczy, mieszczącej się na wysokości 3 metrów wychodząc z pozycji przysiadu, następnie złapanie piłki w górnej fazie wyprostu nóg i powrót do przysiadu;
- *box jumps* - naskok obiema nogami na skrzynię o wysokości 60 cm, następnie zeskok obiema nogami na podłoże;
- *push presses* - wypychanie sztangi nad głowę, wychodząc z lekko ugiętych kolan, w przerwach między wypychaniem sztanga znajdowała się na wysokości barków. Po

osiągnięciu maksymalnej wysokości sztangi należało zablokować ramiona w barkach oraz łokciach;

- *rowing* - ćwiczenie wykonywane na ergometrze wioślarskim

4.3. Ocena wydolności aerobowej

Ocena poziomu wydolności aerobowej sportowców, oparta m.in. na oznaczeniu ich maksymalnego poboru tlenu (VO_2max) i progu wentylacyjnego (VT), zostanie przeprowadzona za pomocą próby czynnościowej o wzrastającej intensywności, na cykloergometrze Kettler X1 (Niemcy), z uwzględnieniem zaleceń wykonywania tego typu testu (Winter i wsp., 2009). Wysiłek testowy będzie rozpoczynany z częstotliwością obrotów 70±3 RPM/min i obciążeniem początkowym, wynoszącym 100 W, które co 1,5 minuty będzie zwiększane o 25 W. Test wysiłkowy zostanie prowadzony do odmowy jego dalszego kontynuowania, niemożności utrzymania wyznaczonej częstotliwości obrotów na cykloergometrze lub braku wzrostu poboru tlenu, które to elementy świadczyć będą o osiągnięciu indywidualnego maksymalnego obciążenia wysiłkowego.

W trakcie prowadzenia testów czynnościowych rejestrowane będą wskaźniki fizjologiczno-metaboliczne z użyciem przenośnego ergospirometru $K4b^2$ (Cosmed, Włochy) i programu komputerowego COSMED CPET Software Suite.

4.3.1. Wyznaczenie maksymalnego poboru tlenu

Wyznaczenie maksymalnego poboru tlenu w trakcie testu czynnościowego, ze wzrastającym obciążeniem zostanie wykonane w oparciu o analizę zmian poboru tlenu, w zależności od intensywności wysiłku fizycznego. Wykonywany pomiar poboru tlenu w tym urządzeniu będzie opierał się na standardowej metodzie Douglasa (Bruce i wsp., 1963). Osiągnięcie maksymalnego wysiłku będzie interpretowane w momencie braku przyrostu poboru tlenu (VO_2) i częstości skurczów serca (HR) i/lub odmowy zawodnika do kontynuowania wysiłku.

4.3.2. Wyznaczenie progu wentylacyjnego

Wyznaczenie progu wentylacyjnego, jako markera progu przemian beztlenowych zostanie wykonane metodą „*V-slope*”, opierającą się na analizie regresji liniowej krzywej rosnącego wydzielania CO_2 w stosunku do krzywej rosnącego poboru O_2 i wyznaczeniu punktu, w którym obserwowany od początku testu prostoliniowy przebieg zależności VCO_2 i VO_2, zacznie intensywnie wzrastać, z powodu nieproporcjonalnego przyrostu wydalanego CO_2 w stosunku do poboru O_2 (Beaver i wsp., 1986).

Do scharakteryzowania progu wentylacyjnego (VT), wyznaczonego w trakcie testu wysiłkowego o wzrastającej intensywności, wykorzystane zostaną wskaźniki, jak: czas osiągnięcia progu wentylacyjnego (T_{VT}), obciążenie progowe (W_{VT}) oraz progową częstość skurczów serca (HR_{VT}).

4.4. Analiza biochemiczna wybranych markerów we krwi

W niniejszych badania zostania wykonana analiza biochemiczna jednych z najczęściej stosowanych w badaniach z udziałem sportowców markerów adaptacji i homeostazy. Krew włośniczkowa z opuszki palca będzie pobierana bezpośrednio przed oraz 3 minuty po zakończeniu prób wysiłkowych (test progresywny i FGB). Aktywność enzymów: kinazy kreatynowej (CK) i dehydrogenazy mleczanowej (LDH), a także stężenie glukozy, pirogronianu i mleczanu będzie oceniane na podstawie analizy ilościowej we krwi sportowców, z wykorzystaniem komercyjnych testów diagnostycznych.

5. Bibliografia

1. Beaver WL, Wasserman K, Whipp BJ. A new method for detecting the anaerobic threshold by gas exchange. J Appl Physiol., 1986, 60(6), 2020-2027.
2. Bentzur K.M., Kravitz L., Lockner D.W. Evaluation of the BOD POD for estimating percent body fat in collegiate track and field female athletes: a comparison of four methods. J. Strength Cond. Res., 2008, 22, 1985–1991.
3. Bruce RA, Blackmon JR, Jones JW, Strait G. Exercise testing in adult normal subjects and cardiac patients. Pediatrics, 1963, 32(Suppl.), 742-756.

4. Carr AJ, Hopkins WG, Gore CJ. Effects of acute alkalosis and acidosis on performance: a meta-analysis. Sports Med. 2011;41(10):801–14.
5. CDC - Centers for Disease Control and Prevention: National Health and Nutrition Examination Survey (NHANES). Anthropometry procedures manual, 2007. www.cdc.gov/nchs/data/nhanes/nhanes_07_08/manual_an.pdf
6. Coso JD, Hamouti N, Agudo-Jimenez R, Mora-Rodriguez R. Restoration of blood pH between repeated bouts of high-intensity exercise: effects of various active-recovery protocols. Eur J Appl Physiol. 2010;108:523–532.
7. Goins J.M.: Physiological and performance effects of crossfit. PhD dissertation, Tuscaloosa, Alabama, 2014.
8. Kahlea LE, Kellyb PV, Eliota KA, Weissa EP. Acute sodium bicarbonate loading has negligible effects on resting and exercise blood pressure but causes gastrointestinal distress. Nutr Res. 2013;33(6):479–486.
9. Kyle UG, Bosaeus I, De Lorenzo AD, Deurenberg P, Elia M, Gómez JM, et al. Bioelectrical impedance analysis-part I: review of principles and methods. Clin Nutr., 2004, 23(5), 1226-1243.
10. Lukaski HC, Johnson PC. Assessment of fat-free mass using bio-electrical impedance measurement of the human body. Am J Clin Nutr., 1985, 41(4), 810-817.
11. Peart DJ, Siegler JC, Vince RV. Practical recommendations for coaches and athletes: a meta-analysis of sodium bicarbonate use for athletic performance. J Strength Cond Res. 2012;26(7):1975–83.
12. Siegler JC, Marshall PW, Bishop D, Shaw G, Green S. Mechanistic insights into the efficacy of sodium bicarbonate supplementation to improve athletic performance. Sports Med Open. 2016;2(1):41.
13. Winter EM, Jones AM, Davison RCR, Bromley PD, Mercer T. Sport and exercise physiology testing guidelines: vol. 1 Sport testing: The British Association of Sport and Exercise Sciences. Taylor and Francis e-Library, Abingdon 2009.
