# Supplementary material for: The effect of chronic progressive-dose sodium bicarbonate ingestion on CrossFit-like performance: A double-blind, randomized cross-over trial
Source: PLoS One. 2018 May 17;13(5):e0197480. doi: 10.1371/journal.pone.0197480 (PMC5957406; doi:10.1371/journal.pone.0197480)
Supplement: S2 File — (PDF) [file pone.0197480.s002.pdf]

Poznan University of Life Sciences
Faculty of Food Science and Nutrition
Department of Hygiene and Human Nutrition
Department Chair: Professor Jan Jeszka
Principal Investigator: Krzysztof Durkalec-Michalski
Wojska Polskiego 31, 60-637 Poznan, Poland
Phone/Fax: (+48 61) 848 73 32

STUDY PROTOCOL

THE EFFECT OF SODIUM BICARBONATE SUPPLEMENTATION IN ATHLETES

1. Introduction

High-intensity exercise results in the accumulation of lactate and hydrogen ions (H^+) as a consequence of intensified anaerobic glycolysis when limited amounts of oxygen are available to the working muscle cells (Coso et al., 2010). The increasing intracellular acidosis is implicated as one of many factors contributing to the perception of fatigue (Siegler et al., 2016). During high-intensity exercise, intramuscular acidity is regulated by different buffering systems and bicarbonate (HCO_3^-) in particular is the major contributor to the buffering system in the blood. In theory, increasing HCO_3^- concentration through it supplementation could augment exercise performance. Indeed, in a meta-analysis the oral ingestion of sodium bicarbonate (SB) resulted in a moderate performance augmentation of 1.7 % in a single 1-minute sprint with a further ~1 % improvement in repeated sprint performance (Carr et al., 2011). Furthermore, in another meta-analysis authors confirmed favorable effect of SB supplementation on physical performance and concluded that individual protocol of bicarbonate supplementation should be developed to avoid adverse effects (Peart i wsp., 2012). Since CrossFit is a high-intensity training, athletes could, in theory, benefit largely from the supplementation of SB. However, no studies have examined the effect of SB in CrossFit athletes so far.

2. Supplementation regimen

This was a crossover, randomized, double-blinded study.
Group taking oral sodium bicarbonate supplementation: the experimental procedure for each athlete will include a 10-day $NaHCO_3$ supplementation in a progressive-dose regimen in order to reduce the likelihood of gastrointestinal side effects (from 37.5 to 150 mg · kg^{-1}) (Kahlea et

al., 2013). $NaHCO_3$ will be administered in the form of unmarked disk-shaped tablets (Alkala T, SANUM, Poland). The content of $NaHCO_3$ in one tablet will be 1 g. The tablets will be ingested with at least 250 mL of water and would be either swallowed or dissolved in the mouth. On training days the supplements will be taken in the morning, in the evening and 1.5 hours before training session. On rest days the supplements will be taken in the morning, in the afternoon and in the evening. Between the 10-day $NaHCO_3$ and PLA or a PLA and $NaHCO_3$ treatments, a 14-day washout period will be introduced.

Group taking oral supplementation with placebo (maltodextrin, NaCl): the experimental procedure for each athlete will include a 10-day placebo administration. Placebo will be ingested in a similar tablet form with at least 250 mL of water. On training days the supplements will be taken in the morning, in the evening and 1.5 hours before training session. On rest days the supplements will be taken in the morning, in the afternoon and in the evening. Between the 10-day $NaHCO_3$ and PLA or a PLA and $NaHCO_3$ treatments, a 14-day washout period will be introduced.

3. Participants

Approximately 30 people (males and females), aged 20-43 years will participate in the study. The participants will recreationally and regularly train CrossFit. The criteria for qualifying for the study will include, among others, good health condition, a valid and up-to-date medical certificate confirming the athlete's ability to practice sports, at least 2 years of CrossFit training experience, and a minimum of 4 workout sessions a week (minimum 3 in the practiced CrossFit discipline).

4. Methods

4.1. Evaluation of anthropometric indices and body composition

4.1.1. Evaluation of body mass and height

Evaluation of body mass and height in the examined group of athletes will be conducted using medical scales certified for compliancy with the standard and medical directives, legalized and coupled with a WPT 60/150 OW anthropometer by RADWAG® (Polska).

Analyses of body mass and height will be conducted in the morning using all recommended measurement procedures (CDC 2007).

4.1.2. Evaluation of body composition by electrical bioimpedance

Analysis of body composition by electrical bioimpedance will be conducted using a 4-electrode BIA 101S analyser by AKERN-RJL (Italy) and the Bodygram 1.31 computer software by AKERN-RJL (Italy).

Body composition will be measured strictly following the recommended measurement conditions, i.e. in the morning hours, following overnight fasting, in subjects lying in a supine position, and with the recommended application of measuring electrodes (Kyle et al., 2004; Lukaski and Johnson 1986). Athletes will be also instructed to abstain from drinking coffee, strong tea, caffeine-containing products, and alcohol for at least 24 h before the test, as well as to refrain from physical exercise for a minimum of 18 h before measurements.

4.1.3. Body composition measurement by air displacement plethysmography

Fat and free fat mass will be assessed based on air displacement plethysmography using the Bod Pod® (Cosmed, Italy). Once the body density will be determined, the body fat and free fat mass will be calculated using the Siri equation. Thoracic lung volume will be estimated using the Bod Pod® software. During the measurement the participants are going to wear only a swimsuit and an acrylic swim cap (Bentzur et al. 2008).

4.2. Specific physical fitness test "*Fight Gone Bad*" (FGB)

In order to assess CrossFit performance the FGB workout, which can be used as a physical fitness test as well, was performed (Goins, 2014). The FGB will consist of 5 multi-joint exercises (wall ball shots, sumo deadlift high-pulls, box jumps, push presses, and rowing) repeated in 3 rounds. Each FGB test will be performed in the afternoon hours the next day after ICT. During one round the participants will perform five different 1-min exercises (wall ball shots, sumo deadlift high-pulls, box jumps, push presses and rowing). The participants will be asked to do as many repetitions of each single exercise as they would during 1 minute, and then immediately will move to the next exercise. Rounds will be separated by one-minute breaks. The entire FGB test will be video recorded so as to allow precise counting of all

technically-well performed repetitions. Repetitions will be counted only if the participant complete a full range of motion required for each exercise. The whole FGB workout will last for 17 min (3 rounds x 5 min and 2 breaks x 1 min; single 5-min round: 5 exercises x 1 min).

4.3. Evaluation of aerobic capacity of athletes

Evaluation of aerobic capacity of athletes, based on e.g. the determination of their maximal oxygen uptake (VO_2max) and ventilatory threshold (VT), will be conducted using an increasing intensity exercise test (ICT) on a Kettler X1 cycloergometer (Germany) following recommendations for such tests (Winter et al., 2009). The exercise test will be started at rotation frequency of 70±3 RPM/min and initial load of 100 W, which will be increased by 25 W at every 1.5 minutes. The exercise test will be conducted until the participant refuses to continue, it is impossible to maintain the set rotation frequency on the cycloergometer or a lack of increase in oxygen uptake, which will indicate that the individual maximal exercise load has been reached.

During the exercise tests physiological and metabolic indices will be recorded using a portable K4b^2 ergospirometer (Cosmed, Italy), and COSMED CPET Software Suite.

4.3.1. Determination of maximal oxygen uptake

Determination of maximal oxygen uptake during the exercise test with an increasing load will be conducted based on the analysis of changes in oxygen uptake, depending on the intensity of physical exercise. The recorded measurement of oxygen uptake in that apparatus will be based on the standard method proposed by Douglas (Bruce et al., 1963). Reaching maximal exercise will be interpreted as the moment of a lack of increment in oxygen uptake (VO_2) and heart rate (HR) and/or the participant's refusal to continue exercise.

4.3.2. Determination of ventilatory threshold

Determination of ventilatory threshold as a marker of the anaerobic metabolism threshold will be conducted by the *V-slope* method, based on the analysis of linear regression of the curve of increasing CO_2 release in relation to the curve of increasing O_2 uptake and the determination of a point, at which the straight linear course of the dependence between VCO_2 and VO_2,

observed from the beginning of the test, starts to intensively increase due to a disproportional increment in the exhaled CO_2 in relation to O_2 uptake (Beaver et al., 1986).

The ventilatory threshold (VT) determined during the increasing intensity exercise test will be characterized based on such indices as time to reach ventilatory threshold (T_{VT}), threshold load (W_{VT}) and threshold heart rate (HR_{VT}).

4.4. Biochemical analyses of selected blood markers

The most widely used markers of adaptation and homeostasis in studies involving athletes will be applied in this investigation. Fingertip blood samples will be taken immediately pre exercise and 3 minutes post exercise (ICT or FGB). The activity of the creatine kinase (CK) and lactate dehydrogenase (LDH) enzymes, and the concentration of glucose, pyruvate and lactate will be assessed based on a quantitative analysis of the blood of the athletes using commercial diagnostic tests.

References

1. Beaver WL, Wasserman K, Whipp BJ. A new method for detecting the anaerobic threshold by gas exchange. J Appl Physiol., 1986, 60(6), 2020-2027.
2. Bentzur K.M., Kravitz L., Lockner D.W. Evaluation of the BOD POD for estimating percent body fat in collegiate track and field female athletes: a comparison of four methods. J. Strength Cond. Res., 2008, 22, 1985–1991.
3. Bruce RA, Blackmon JR, Jones JW, Strait G. Exercise testing in adult normal subjects and cardiac patients. Pediatrics, 1963, 32(Suppl.), 742-756.
4. Carr AJ, Hopkins WG, Gore CJ. Effects of acute alkalosis and acidosis on performance: a meta-analysis. Sports Med. 2011;41(10):801–14.
5. CDC - Centers for Disease Control and Prevention: National Health and Nutrition Examination Survey (NHANES). Anthropometry procedures manual, 2007. www.cdc.gov/nchs/data/nhanes/nhanes_07_08/manual_an.pdf
6. Coso JD, Hamouti N, Agudo-Jimenez R, Mora-Rodriguez R. Restoration of blood pH between repeated bouts of high-intensity exercise: effects of various active-recovery protocols. Eur J Appl Physiol. 2010;108:523–532.
7. Goins J.M.: Physiological and performance effects of crossfit. PhD dissertation, Tuscaloosa, Alabama, 2014.

8. Kahlea LE, Kellyb PV, Eliota KA, Weissa EP. Acute sodium bicarbonate loading has negligible effects on resting and exercise blood pressure but causes gastrointestinal distress. Nutr Res. 2013;33(6):479–486.
9. Kyle UG, Bosaeus I, De Lorenzo AD, Deurenberg P, Elia M, Gómez JM, et al. Bioelectrical impedance analysis-part I: review of principles and methods. Clin Nutr., 2004, 23(5), 1226-1243.
10. Lukaski HC, Johnson PC. Assessment of fat-free mass using bio-electrical impedance measurement of the human body. Am J Clin Nutr., 1985, 41(4), 810-817.
11. Peart DJ, Siegler JC, Vince RV. Practical recommendations for coaches and athletes: a meta-analysis of sodium bicarbonate use for athletic performance. J Strength Cond Res. 2012;26(7):1975–83.
12. Siegler JC, Marshall PW, Bishop D, Shaw G, Green S. Mechanistic insights into the efficacy of sodium bicarbonate supplementation to improve athletic performance. Sports Med Open. 2016;2(1):41.
13. Winter EM, Jones AM, Davison RCR, Bromley PD, Mercer T. Sport and exercise physiology testing guidelines: vol. 1 Sport testing: The British Association of Sport and Exercise Sciences. Taylor and Francis e-Library, Abingdon 2009.
