# Supplementary material for: The effect of chronic progressive-dose sodium bicarbonate ingestion on CrossFit-like performance: A double-blind, randomized cross-over trial
Source: PLoS One. 2018 May 17;13(5):e0197480. doi: 10.1371/journal.pone.0197480 (PMC5957406; doi:10.1371/journal.pone.0197480)
Supplement: S1 Table — (PDF) [file pone.0197480.s003.pdf]

S1 Table. Baseline characteristics of the participating athletes (*N* = 21 subjects).

	SB_{pre}			PLA_{pre}			*p value* [a] Baseline (SB vs. PLA)
Age (yr)			32 ± 5				-
Body heigh (cm)			174 ± 8				-
Body mass (kg)	73.0	±	14.0	73.2	±	13.8	0.455
FM (kg)	13.6	±	4.9	13.9	±	4.9	0.075
FFM (kg)	58.4	±	13.4	59.3	±	13.7	0.140
TBW (L)	44.7	±	10.2	44.3	±	9.6	0.509

Data are mean ± SD. SB, sodium bicarbonate; PLA, placebo; FM, fat mass; FFM, fat free mass; TBW, total body water content. [a] Depending on the data distribution (normal; not normal): one side paired t-Student test or the Wilcoxon test.
