# Supplementary material for: The effect of chronic progressive-dose sodium bicarbonate ingestion on CrossFit-like performance: A double-blind, randomized cross-over trial
Source: PLoS One. 2018 May 17;13(5):e0197480. doi: 10.1371/journal.pone.0197480 (PMC5957406; doi:10.1371/journal.pone.0197480)
Supplement: S3 Table — (PDF) [file pone.0197480.s005.pdf]

S3 Table. Gastrointestinal side effects during SB and PLA supplementation.

	SB trial					PLA trial				
	**day 1**	**day 3**	**day 5**	**day 8**	**day 10**	**day 1**	**day 3**	**day 5**	**day 8**	**day 10**
Stomach problems	1.0 ± 1.0	1.0 ± 1.0	1.0 ± 1.2	0.5 ± 0.9	0.3 ± 0.7	1.0 ± 1.3	0.5 ± 0.8	0.7 ± 1.0	0.5 ± 0.9	0.4 ± 0.7
Nausea	0.3 ± 0.6	0.3 ± 0.6	0.2 ± 0.5	0.1 ± 0.5	0.1 ± 0.3	0.2 ± 0.4	0.4 ± 0.6	0.2 ± 0.5	0.3 ± 0.6	0.2 ± 0.5
Dizziness	0.1 ± 0.3	0.2 ± 0.4	0.1 ± 0.3	0.0 ± 0.2	0.0 ± 0.0	0.2 ± 0.4	0.1 ± 0.4	0.2 ± 0.4	0.1 ± 0.3	0.1 ± 0.3
Headache	0.6 ± 0.5	0.6 ± 1.0	0.6 ± 1.1	0.4 ± 0.8	0.5 ± 0.9	0.5 ± 0.9	0.5 ± 0.9	0.3 ± 0.8	0.2 ± 0.6	0.3 ± 0.8
Flatulence	0.3 ± 0.6	0.1 ± 0.5	0.2 ± 0.4	0.2 ± 0.4	0.2 ± 0.4	0.2 ± 0.5	0.2 ± 0.4	0.3 ± 0.5	0.2 ± 0.4	0.1 ± 0.4
Urge to urinate	0.3 ± 0.6	0.1 ± 0.4	0.4 ± 0.5	0.1 ± 0.4	0.1 ± 0.3	0.2 ± 0.4	0.3 ± 0.6	0.2 ± 0.4	0.2 ± 0.4	0.1 ± 0.4
Urge to defecate	0.4 ± 0.7	0.4 ± 0.7	0.3 ± 0.6	0.1 ± 0.4	0.4 ± 0.9	0.5 ± 1.0	0.5 ± 1.0	0.5 ± 1.0	0.5 ± 0.9	0.2 ± 0.5
Belching	0.4 ± 1.0	0.5 ± 0.8	0.3 ± 0.6	0.2 ± 0.5	0.3 ± 0.6	0.2 ± 0.5	0.1 ± 0.4	0.2 ± 0.4	0.4 ± 0.7	0.1 ± 0.5
Heartburn	0.3 ± 0.6	0.3 ± 0.6	0.5 ± 0.9	0.1 ±0.5	0.1 ± 0.4	0.4 ± 0.7	0.2 ± 0.6	0.0 ± 0.2	0.1 ± 0.5	0.2 ± 0.6
Bloating	0.4 ± 0.9	0.3 ± 0.6	0.6 ± 1.1	0.4 ± 0.8	0.2 ± 0.6	0.5 ± 0.9	0.4 ± 0.9	0.3 ± 0.7	0.4 ± 0.7	0.4 ± 0.8
Stomach cramps	0.2 ± 0.4	0.1 ± 0.5	0.3 ± 0.8	0.2 ± 0.5	0.2 ± 0.8	0.3 ± 0.6	0.2 ± 0.5	0.5 ± 0.9	0.4 ± 0.8	0.3 ± 0.8
Intestinal cramps	0.4 ± 0.7	0.2 ± 0.4	0.1 ± 0.3	0.0 ± 0.2	0.1 ± 0.3	0.2 ± 0.2	0.1 ± 0.5	0.2 ± 0.4	0.2 ± 0.4	0.2 ± 0.5
Urge to vomit	0.0 ± 0.0	0.0 ± 0.0	0.1 ± 0.3	0.0 ± 0.0	0.0 ± 0.0	0.0 ± 0.2	0.0 ± 0.2	0.0 ± 0.0	0.1 ± 0.3	0.1 ± 0.3
Vomiting	0.0 ± 0.0	0.0 ± 0.0	0.0 ± 0.0	0.0 ± 0.0	0.0 ± 0.0	0.0 ± 0.0	0.0 ± 0.0	0.0 ± 0.0	0.0 ± 0.0	0.0 ± 0.0
Diahrrea	0.5 ± 0.9	0.3 ± 0.6	0.3 ± 0.6	0.3 ± 0.8	0.2 ± 0.7	0.4 ± 0.9	0.3 ± 0.8	0.5 ± 1.0	0.3 ± 0.6	0.2 ± 0.4
Side ache left	0.0 ± 0.0	0.0 ± 0.0	0.1 ± 0.3	0.0 ± 0.2	0.0 ± 0.2	0.0 ± 0.2	0.0 ± 0.2	0.0 ± 0.0	0.0 ± 0.2	0.0 ± 0.0
Side ache right	0.0 ± 0.0	0.0 ± 0.0	0.0 ± 0.2	0.0 ± 0.0	0.1 ± 0.3	0.0 ± 0.0	0.0 ± 0.0	0.0 ± 0.0	0.0 ± 0.0	0.0 ± 0.0
Muscle cramps	0.4 ± 0.8	0.1 ± 0.4	0.2 ± 0.5	0.2 ± 0.6	0.2 ± 0.6	0.5 ± 1.0	0.5 ± 0.9	0.2 ± 0.6	0.2 ± 0.6	0.2 ± 0.5
Cold shivering	0.0 ± 0.0	0.0 ± 0.0	0.0 ± 0.2	0.0 ± 0.0	0.0 ± 0.0	0.0 ± 0.0	0.0 ± 0.0	0.0 ± 0.0s	0.0 ± 0.0	0.0 ± 0.2

Data are mean ± SD. SB, sodium bicarbonate; PLA, placebo.
