# Supplementary material for: The effect of chronic progressive-dose sodium bicarbonate ingestion on CrossFit-like performance: A double-blind, randomized cross-over trial
Source: PLoS One. 2018 May 17;13(5):e0197480. doi: 10.1371/journal.pone.0197480 (PMC5957406; doi:10.1371/journal.pone.0197480)
Supplement: S4 Table — (PDF) [file pone.0197480.s006.pdf]

S4 Table. Heart rates in *Fight Gone Bad* test.

	**SB_{pre}**	**SB_{post}**	**PLA_{pre}**	**PLA_{post}**
Round 1 (bpm)	172 ± 11	166 ± 10	167± 11	168 ± 12
Break 1 (bpm)	163 ± 11	156 ± 11	161 ± 10	159 ± 11
Round 2 (bpm)	173 ± 9	171 ± 10	171 ± 9	171 ± 10
Break 2 (bpm)	166 ± 11	163 ± 10	165 ± 10	165 ± 9
Round 3 (bpm)	174 ± 9	172 ± 8	171 ± 6	173 ± 9
Break 3 (bpm)	167 ± 12	166 ± 8	166 ± 8	165 ± 16
All 3 rounds (bpm)	172 ± 9	169 ± 10	169 ± 9	169 ± 10

Data are mean of average HR ± SD. HR, heart rate; SB, sodium bicarbonate; PLA, placebo.
